# Editorial: Community series in microbiological safety and quality aspects of fermented dairy products, volume II

**DOI:** 10.3389/fmicb.2023.1182373

**Published:** 2023-03-21

**Authors:** Uelinton Manoel Pinto, Juliano De Dea Lindner

**Affiliations:** ^1^Food Research Center, Department of Food and Experimental Nutrition, Faculty of Pharmaceutical Sciences, University of São Paulo (USP), São Paulo, Brazil; ^2^Department of Food Science and Technology, Food Technology and Bioprocess Research Group, Federal University of Santa Catarina (UFSC), Florianópolis, Brazil

**Keywords:** raw milk cheeses, food safety, foodborne pathogens, bioactive peptides, ripening

Factors such as milk type and production processes affect the quality and safety of fermented dairy products. The microbial composition and dynamics during fermentation and ripening are crucial for achieving desired properties, including sensory and health-related aspects such as probiotic potential and the production of beneficial metabolites. However, there is limited knowledge about microbial diversity in many traditional regional products, and new technologies such as genomic approaches and metabolomics have further advanced our understanding over microbial cultivation techniques.

This Research Topic is the second volume of the “*Community series in microbiological safety and quality aspects of fermented dairy products*”. This series aimed to enhance our understanding of the microbial quality and safety of fermented dairy products by collecting additional studies on the impact of the microbiota on the quality and safety of these foods. This effort resulted in a collection of seven articles that focused on the microbiological safety and quality of different fermented dairy products.

Due to the importance of *Listeria monocytogenes* in food safety, Ciccio et al. studied the survival of this pathogen at various storage temperatures (37°C, 12°C, and 4°C) on three food packaging materials commonly used in dairy products: polyethylene-coated parchment; polyethylene-coated nylon and greaseproof paper. The authors contaminated these materials with a pool of five *L. monocytogenes* strains with initial populations ranging between 2.5 and 2.7 log CFU/cm^2^ suspended in a cheese homogenate, presenting an indigenous microbial population. They periodically analyzed samples to determine the total viable count and the pathogen population along 56 days of storage. The results showed that the pathogen was rapidly inactivated at 37°C, with no detectable levels on all materials by the seventh day of storage. This may be explained by the antagonistic effect of the mesophilic lactic acid bacteria (LAB) of the cheese at this elevated temperature. However, all three types of packaging materials harbored the pathogen for the entire experiment duration at 4°C and 12°C. The data clearly showed that the pathogen's survival in packaging materials used in dairy products is higher at 4 and 12°C than at 37°C, which may be explained by the extraordinary ability of *L. monocytogenes* to survive under cold storage. Additionally, irrespective of the storage temperature, the study found that packaging materials most and least conducive to the survival of *L. monocytogenes* in cheese homogenate were greaseproof paper and polyethylene-coated nylon, respectively. Packaging materials, therefore, can be considered potential sources of cross-contamination during processing and food handling. The authors suggested that further studies are necessary to evaluate a broader range of food packaging materials concerning storage and distribution conditions.

In the dairy industry, microbial testing of indicator organisms typically includes the enumeration of total mesophilic bacteria, coagulase-positive staphylococci, and Enterobacteriaceae. Mesophilic organisms, as well as Enterobacteriaceae, are common microorganisms found in many types of dairy products. However, high loads of Enterobacteriaceae may indicate poor microbiological quality of these foods. Additionally, due to the absence of automation and control over environmental and processing parameters in small-scale artisanal cheese production facilities, microbial risk contamination may increase. Microbial testing is even more critical for cheeses that pose a higher risk to consumers, such as those with short ripening times and higher water content, including those classified as “soft” cheeses. Following this rationale, Pasquali et al. studied the physicochemical and microbial parameters of a spreadable soft cheese with no rind produced in an Italian artisanal factory, focusing on inter- and intra-batch variability during one year of investigation. A large number of samples were evaluated, including milk, calf rennet, environmental samples such as those obtained from walls and drains in production, ripening, and packaging areas, as well as from cheeses stored for 15 days at constant temperatures of 2°C and 8°C and in a dynamic temperature profile of 2°C for 5 days and 8°C for 10 days. The results showed that the pH and water activity of the cheese did not change among different batches. Conversely, there was a high inter-batch variability for LAB and total bacteria count (TBC) in cheese at the end of production. As expected, cheeses stored at 8°C had higher loads of Enterobacteriaceae, LAB, and TBC over the shelf life. Of the Enterobacteriaceae isolates identified, most belonged to *Klebsiella oxytoca* and *K. pneumoniae, Enterobacter cloacae, Hafnia alvei*, and *Citrobacter freundii*. The authors argued that there is a need to standardize the microbial quality of cow milk and to implement proper hygienic procedures for cleaning and disinfection, especially in production and ripening rooms. They developed generalized mixed models, which indicated that drains in the warm room, where the recently produced cheese is kept for 1.5 h before going to cold rooms for ripening, and packaging area were associated with higher loads of TBC and Enterobacteriaceae in the cheese. The study did not detect Shiga-toxin-producing *Escherichia coli* (STEC) and *L. monocytogenes*, but a few *Staphylococcus aureus* isolates and one *Salmonella* Pullorum was isolated during storage and processing, respectively. It is important to mention that the sole detection *of S. aureus* in artisanal cheese does not pose a health risk, especially because many countries have limits ranging from 3.0 to 5.0 log CFU/g of coagulase-positive staphylococci in these products. On the other hand, the detection of *Salmonella* is a food safety concern. To conclude, Pasquali et al. suggested that additional studies are needed to investigate the potential pathogenicity and antimicrobial resistance of the identified Enterobacteriaceae species in artisanal cheeses.

Still, on the food safety aspect of artisanal cheeses, Cortimiglia et al. used Whole Genome Sequencing (WGS) to characterize STEC strains obtained from semi-hard raw milk cheese. The authors argue that WGS is a highly effective method for identifying and characterizing pathogenic organisms, aiding surveillance and microbiological risk assessment while also providing high-quality information. Of notice, STEC is a significant contributor to foodborne diseases, and in recent years, an increasing number of infections have been linked to the consumption of raw and pasteurized milk cheeses. This is not surprising since cattle are asymptomatic natural reservoirs of STEC, serving as vehicles of infections to humans through direct contact or the consumption of contaminated foods. Notably, the production and ripening of dairy products made from unpasteurized milk may not completely eliminate certain strains of STEC, as demonstrated in some studies. Thus, Cortimiglia et al. evaluated the pathogenic potential of STEC strains by analyzing their main genomic features. Sample collection took place over 10 months as part of a food company's internal quality check program and let to the isolation of seven strains from four *stx*-positive enrichments. The genome analysis revealed the presence of serotypes O174:H2 and O116:H48, carrying at least one *stx* gene but being negative for the *eae* gene. The virulence gene pattern was homogenous among the serogroups and included adherence factors, enterohemolysin, serum resistance, cytotoxin-encoding genes, and the locus of adhesion and autoaggregation pathogenicity islands (LAA), which is commonly found in Locus of Enterocyte Effacement (LEE)-negative STEC strains. There was a clear indication of genome plasticity as prophagic sequences carrying *stx* genes and plasmid replicons were detected. Despite this, antibiotic-resistance genes were not found in the analyzed genomes. The study provided insights into STEC monitoring in raw milk cheeses, highlighting the important role of WGS in typing unknown isolates. The authors argued that continuous monitoring is essential to protect human health and increase our understanding of STEC genetic features, which will aid developing mitigation strategies at the farm and cheese manufacturing facilities to ensure the safety of the final product.

Enterococci (a diverse group of Gram-positive LAB) are ubiquitous microorganisms. They could constitute a health threat since they produce biogenic amines (BAs) that can lead to intoxication, such as consuming contaminated fermented food. However, they still play a role in producing fermented foods, and some strains have been proposed as probiotics (Baccouri et al.). Furthermore, these bacteria improve the safety of dairy products; for example, *Enterococcus faecium* and *Enterococcus faecalis* produce bacteriocins against pathogens such as *L. monocytogenes*. Enterococci have also emerged as a critical hospital-acquired pathogen by acquiring antimicrobial resistance. Bacteriophages can control bacterial growth in food spoilage contexts. del Rio et al. reported the isolation of *E. faecalis*-infecting bacteriophages from different types of cheese. The isolated phages showed a large diversity regarding the number and origin of strains they infect. Phages' morphological and genomic characterization confirmed families and genera diversity and the number and origin of strains they infect. The results increase the potential arsenal of phages that might be used to control harmful *E. faecalis* in fermented foods in which it may cause the accumulation of BAs.

Baccouri et al. isolated from traditional Tunisian fermented dairy products (Testouri cheese and Rigouta, a close relative to the Italian Ricotta), *E. faecalis* identified by matrix-assisted laser desorption-ionization time of flight mass spectrometry (MALDI TOF-MS) and species-specific PCR. Two isolates (OB15 and OB14) were evaluated for probiotic properties and compared to *E. faecalis* Symbioflor 1 clone DSM 16431 as reference. Cluster analysis by the MALDI TOF-MS biotype system showed that *E. faecalis* OB15 was more closely related to the Symbioflor 1 probiotic strain than OB14, confirmed by WGS using the average nucleotide identity (ANI) and Genome-to-Genome Hybridization similarity methods. In the minimum inhibitory concentration test, *E. faecalis* OB14 and OB15 were sensitive to ampicillin and vancomycin. WGS also showed tetracycline resistance and cytolysin genes in the OB14 strain. According to the results, the OB15 strain demonstrated the potential to be further studied for future development as a food or feed industry probiotic.

Raw milk is considered the major source of *Mycobacterium avium* subsp. *paratuberculosis* (MAP) exposure for humans. Barsi et al. determined the inactivation kinetics (thermal death time values) of MAP in curd used to produce pasta-filata cheese (e.g., Mozzarella) for the first time in state of art. During the Mozzarella cheese making, the curd is stretched (from the Italian “filare”) by adding hot water. This is a critical processing step for the inactivation of MAP. In the study, the milk was challenged with a mix of MAP strains inoculum. Samples of contaminated curd were treated separately at six different temperatures, from 60°C to 75°C, in a water bath. MAP survival was evaluated by the plate count method, and inactivation parameters were estimated directly in the treated curd. D-values increased from 0.15 min (75) to 4.22 min (60) with a 10.2°C z-value. The work results corroborated to predict the binomial Time/Temperature factor needed to obtain a MAP log reduction during the stretching step, to optimize or validate the pasta-filata cheese process, and design thermal treatment defining acceptance limits of critical control points to ensure safety against MAP.

Although the health effects associated with the consumption of long-ripened cheeses has been debated due to high salt and saturated fats, it has been associated with a reduced risk of non-communicable diseases due to the presence of bioactive peptides (BPs) and non-proteolytic acyl derivatives (NPADs). Castellone et al. investigated the presence of BPs and NPADs in Parmigiano Reggiano cheese, a hard-cooked and long-ripened cheese, at different ripening times, for 24 months. They found a small number of known bioactive compounds with antimicrobial, antioxidant, and ACE-inhibitor activities in the cheese samples, with more potential BPs found after simulated gastrointestinal digestion. The study tracked the fate of peptides as quality drivers during cheese ripening to their release as functional compounds after digestion, suggesting that the benefits of moderate but constant cheese consumption may outweigh the risks.

In summary, this Research Topic offered a comprehensive examination of the microbiological safety and quality of several fermented dairy products, bringing together diverse perspectives and research from leading experts. This Research Topic provides valuable information for researchers, food manufacturers, and policymakers to ensure the continued production and consumption of safe and high-quality fermented dairy products. It is also critical to recognize the importance of continuing research efforts in this field to further advance our understanding of these products and to promote the health and well-being of consumers worldwide.

## Author contributions

All authors have made a substantial, direct, and intellectual contribution to the work and approved it for publication.

